# Embedded Disposable Functionalized Electrochemical Biosensor with a 3D-Printed Flow Cell for Detection of Hepatic Oval Cells (HOCs)

**DOI:** 10.3390/genes9020089

**Published:** 2018-02-14

**Authors:** Samar Damiati, Martin Peacock, Stefan Leonhardt, Laila Damiati, Mohammed A. Baghdadi, Holger Becker, Rimantas Kodzius, Bernhard Schuster

**Affiliations:** 1 Department of Biochemistry, Faculty of Science, King Abdulaziz University (KAU), Jeddah 21589, Saudi Arabia; 2 Institute for Synthetic Bioarchitecture, Department of Nanobiotechnology, University of Natural Resources and Life Sciences, 1190 Vienna, Austria; bernhard.schuster@boku.ac.at; 3 Zimmer and Peacock Ltd., Royston SG8 9JL, UK; martinpeacock@zimmerpeacock.com; 4 Institute of Medical and Polymer Engineering, Technical University of Munich (TUM), 85748 Garching, Germany; stefan.leonhardt@tum.de; 5 Centre for Cell Engineering, University of Glasgow, Glasgow G12 8QQ, UK; l.damiati.1@research.gla.ac.uk; 6 Department of Biology, Jeddah University, Jeddah 23218, Saudi Arabia; 7 Research Centre, King Faisal Specialist Hospital & Research Centre, Jeddah 21499, Saudi Arabia; m-baghdadi@hotmail.com; 8 Microfluidic ChipShop Gmbh, 07747 Jena, Germany; hb@microfluidic-chipshop.com; 9 Mathematics and Natural Sciences Department, The American University of Iraq, Sulaimani, Sulaymaniyah 46001, Iraq; kodzius@envirola.com; 10 Materials Genome Institute, Shanghai University, Shanghai 200444, China; 11 Faculty of Medicine, Ludwig Maximilian University of Munich (LMU), 80539 Munich, Germany; 12 Faculty of Medicine, Technical University of Munich (TUM), 81675 Munich, Germany

**Keywords:** 3D-printed flow-cell, hepatic oval cells, cancer diagnosis, multiwall carbon nanotubes

## Abstract

Hepatic oval cells (HOCs) are considered the progeny of the intrahepatic stem cells that are found in a small population in the liver after hepatocyte proliferation is inhibited. Due to their small number, isolation and capture of these cells constitute a challenging task for immunosensor technology. This work describes the development of a 3D-printed continuous flow system and exploits disposable screen-printed electrodes for the rapid detection of HOCs that over-express the OV6 marker on their membrane. Multiwall carbon nanotube (MWCNT) electrodes have a chitosan film that serves as a scaffold for the immobilization of oval cell marker antibodies (anti-OV6-Ab), which enhance the sensitivity of the biomarker and makes the designed sensor specific for oval cells. The developed sensor can be easily embedded into the 3D-printed flow cell to allow cells to be exposed continuously to the functionalized surface. The continuous flow is intended to increase capture of most of the target cells in the specimen. Contact angle measurements were performed to characterize the nature and quality of the modified sensor surface, and electrochemical measurements (cyclic voltammetry (CV) and square wave voltammetry (SWV)) were performed to confirm the efficiency and selectivity of the fabricated sensor to detect HOCs. The proposed method is valuable for capturing rare cells and could provide an effective tool for cancer diagnosis and detection.

## 1. Introduction

Cancer is one of the leading causes of death worldwide, and the number of newly diagnosed patients is expected to rise. Hence, early detection of cancer is sought to increase the chances for successful treatment and survival rates [1,2,3]. Currently, most research efforts in cancer diagnosis focus on developing biosensors with high selectivity and sensitivity, and inexpensive, rapid and easy operation. Current routine techniques to detect cancer include polymerase chain reaction (PCR), immunohistochemistry, and flow cytometry. Although these methods are effective in cancer diagnosis, they are expensive, time-consuming, and require highly skilled operators [3,4,5,6]. By contrast, electrochemical methods offer many advantages compared with other techniques, such as rapidity, low cost, ease of operation, use of small reagent/sample volumes, and, most importantly, high selectivity and sensitivity in the detection of specific antigens [7,8,9]. Several researchers have incorporated electrochemical biosensors in a microfluidic system to perform chemical and biomedical analysis [10,11]. There are many significant contributions of microfluidic technology that enhance clinical and biological assays and that involve the ability to isolate rare cells from blood on the single-cell scale as an alternative to bulk isolation/detection methods. Devices using continuous flow allow the easy control of physical and chemical environments and provide high levels of experimental automation. Moreover, since rare cells are low in number, continuous-flow devices allow samples to be exposed to the sensor surface continuously, which enhances the capability of capturing the rare cells [12,13,14].

Diagnosis of cancer at an early stage is the key aim in the fabrication of novel biosensors to diagnose patients with not only the metastatic disease but also screen patients with relapse after treatment [15,16,17]. In recent years, rare circulating tumor cells (CTCs) have attracted more attention for improving cancer diagnosis and therapy. However, these cells represent a small percentage of the total blood cells in circulation; among >10^9^ red and white blood cells, there are approximately 1–100 CTCs/mL blood [18,19]. Therefore, targeting CTCs and cancer stem cells (CSCs) requires efficient techniques to capture these rare cells and exclude other cell types [20,21,22,23,24]. Capturing CSCs can be achieved by the detection of cell surface biomarkers, such as CD13, CD24, CD44, CD90, CD133, EpCAM, and OV6, that are highly expressed by cancer cells but not by normal cells [25,26]. In hepatocellular carcinoma (HCC), the third leading cause of cancer mortality worldwide, CSCs can be recognized by several surface antigens. Hepatic oval cells (HOCs) are defined as liver stem/progenitor cells in the liver Herring pipe; they are considered one of the most important origins of liver stem cells [27,28]. Among many markers, OV6 is widely chosen as the best marker to capture and quantify HOCs.

High-efficiency fabricated biosensors can be achieved through careful surface architecture design. Nanomaterials such as carbon nanotubes and graphene have been widely exploited in biosensing applications [29,30]. Among these nanostructured materials, multi-wall carbon nanotubes (MWCNTs) can serve as scaffolds that provide a self-supported structure and possess several features that can be functionalized through conjugation for detection purposes. MWCNTs are long, thin, hollow cylinders of carbon with additional graphene tubes around the core. MWCNTs are strong, stiff fibers due to their well-ordered arrangement of carbon atoms linked via sp^2^ bonds [31,32]. These tubes are in high demand for engineering new devices in different fields because of their unique combination of chemical, optical, electrical, mechanical, and magnetic properties. To design sensitive biosensors, MWCNTs offer several advantages including high surface area, promotion of electron transfer reactions between electroactive compounds and electrodes, minimization of fouling of the electrode surface, enhancement of electro-catalytic activity and immobilization of molecules on their surfaces. Modification of MWCNT platforms with a polymer enables the generation of an efficient immobilization matrix for biosensing technology [33,34]. Chitosan (CS) is a naturally occurring polysaccharide that is derived by the partial deacetylation of chitin. Chitosan provides several advantages such as low cost, non-toxicity, biodegradability, biocompatibility and excellent film-forming ability [35,36,37]. Its multiple functional groups and capability to be chemically modified make chitosan a promising matrix for biosensors.

**Figure 1:**
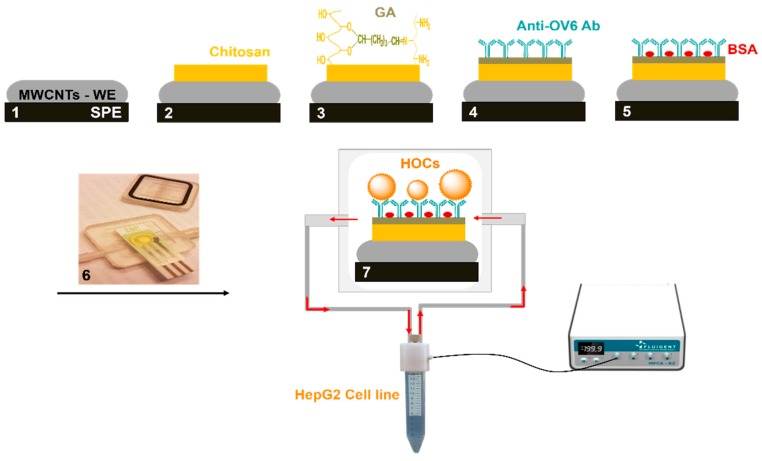
Schematic representation of the fabrication of the electrochemical biosensor for detection of oval cells in the HepG2 cancer cell line in a circulatory platform (not shown to scale). (**1**) Working electrode (WE) of multiwall carbon nanotube (MWCNTs); (**2**) modification of electrode surface with chitosan; (**3**) chitosan film crosslinked with glutaraldehyde (GA); (**4**) immobilization of anti-OV6 antibody onto the activated surface; (**5**) blocking the surface with bovine serum albumin (BSA); (**6**) embedding the developed sensor into the 3D-printed flow cell; and (**7**) connecting to a flow control system to allow for continuous exposure of the cancer cells over the sensor architecture. SPE: screen printed electrode; Ab: antibody.

In the present work, MWCNT electrodes covered by a chitosan film and functionalized with anti-OV6 antibody were developed to target HOCs (Figure 1). The fabricated sensor was embedded with a 3D-printed flow cell to allow for continuous exposure of the cancer cells over the sensor architecture. The electrochemistry of the functionalized sensor was examined using cyclic voltammetry (CV) and square wave voltammetry (SWV). The fabricated system exhibited an excellent cell capture response and may be an effective tool for tumor biomarker detection.

## 2. Materials and Methods 

### 2.1. 3D-Printing Process of the Flow Cell

The flow cell was manufactured using a digital light processing (DLP)-3D-printer (Fab 12, procedure medical GmbH, Dortmund, Germany). Digital light processing is an additive manufacturing method based on selective exposure (UV-light) of a photo polymeric system. Due to the use of a digital mirror device (DMD-chip) a whole layer can be printed at one time. Digital light processing is characterized by its high printing speed, good resolution in x, y and z directions as well as the good surface quality of the printed parts. The used material was an acrylate-based photopolymer optimized for cell-based medical products. The photopolymer is characterized by its good biocompatibility. In several cytotoxicity tests according to German Institute of Standardization—European Standards (DIN-EN) ISO 10993-5, no cytotoxic effects could be observed. The layer thickness during the printing process was 100 µm. After the printing process, the flow cell was cleaned in a 99% isopropanol solution using an ultrasonic bath for 15 min. After the cleaning process, the flow cell was post-cured in a UV-light chamber for 7 min.

### 2.2. Preparation of the Functionalized Chitosan Matrix on the Multiwall Carbon Nanotubes Electrode

As shown in Figure 1, 5 µL of 0.5% *w*/*v* chitosan (Sigma Aldrich, San Luis, MI, USA) in 1% *v*/*v* acetic acid was dropped on an MWCNT electrode and dried at room temperature for 3 h. After rinsing with water, the modified electrode was incubated with 5 µL of 2.5% *v*/*v* glutaraldehyde (GA) (Sigma Aldrich) in phosphate-buffered saline (PBS) for 2 h and then washed with water. Five µL of 200 mg/mL human/rat OV-6 antibody (R&D Systems, Abingdon, UK) in PBS was dropped onto the activated surface and incubated at 4 °C overnight. Excess antibodies were removed by washing with PBS before the modified electrode surface was blocked with 1% bovine serum albumin (BSA) and incubated at room temperature for 90 min to prevent any unspecific adsorption and block any remaining active sites. After a final washing step with PBS, the developed sensors were used immediately or stored at 4 °C.

### 2.3. Contact Angle Measurements

The contact angles of water on the modified film were measured using a goniometer (Easy Drop, Krüss, Hamburg, Germany) at room temperature. Three µL of Milli-Q water was deposited onto the surface, and the angle was measured immediately. All contact angle measurements were repeated at least in triplicate.

### 2.4. Cell Lines and Cell Culture

The liver and breast cancer cells were cultured according to standard mammalian tissue protocols with a sterile technique. Briefly, human liver hepatocellular carcinoma cell line (HepG2) and human breast adenocarcinoma cell line (MCF-7) (American Type Culture Collection) were cultured in DMEM (PAA Laboratories GmbH, Pasching, Austria) supplemented with 10% fetal bovine serum (FBS) or 10 µg/mL insulin, respectively, and a 1% antibiotic/antimycotic solution at 37 °C in 5% CO_2_ and 95% air humidified atmosphere as adherent monolayers in 25 cm^2^ cell culture flasks. After 48 h, the cells were detached from the flask using Trypsin, separated from the medium via centrifugation and counted using an automated cell counter (NanoEntek, Waltham, MA, USA). Trypan blue was used to count and discriminate between viable and non-viable cancer cells. This dye selectively stains non-viable cells and exhibits distinctive blue under the microscope. Briefly, a suspension of cancer cells (HepG2 or MCF-7) in PBS was diluted in Trypan blue solution (0.4%) at a 1:1 ratio. When cell viability was above 85%, the cells were used for further experiments.

### 2.5. Flow Cytometry Analysis 

Flow cytometry was conducted for HepG2 and MCF-7 cancer cells using a Beckman Coulter Elite Xl (Nyon, Switzerland) with OV-6 phycoerythrin monoclonal antibody (R&D Systems). Briefly, both cell lines (1 × 10^6^ cells/mL) were incubated with 10 µL of antibody for 30 min in the dark followed by washing with PBS; the cells were resuspended in fresh PBS and analyzed by flow cytometer immediately. The cells were passed through the laser beam in the flow cytometer at a rate of 10,000 cells/second.

### 2.6. Electrochemical Measurements

The three-electrode system was printed on ceramic substrates with dimensions: L3.4 × W1.0 × H0.05 cm, and three-electrode configuration was incorporated: counter electrode (CE, carbon), reference electrode (RE, silver), and working electrode (WE, MWCNT, 400 µm diameter). All CV and SWV measurements were performed at least in duplicate using a potentiostat (Zimmer and Peacock, Royston, UK). Cyclic voltammetry measurements were recorded for each functionalized layer of the developed sensor after rinsing with PBS. The modified electrodes were embedded into the 3D-printed flow cell, which then connected to a flow control system (Fluigent, Paris, France) that allows cancer cell injection at different concentrations, and SWV measurements were recorded after rinsing with PBS to remove unbound cells.

## 3. Results and Discussion 

### 3.1. Contact Angle Measurements and Surface Sensor Characterization

Measurement of the contact angle between water and the modified surface is typically used as an indicator for surface hydrophilicity/hydrophobicity characteristics. However, the surface wetting properties determine the quality of the fabricated sensor, which affects cell attachment and proliferation.

**Figure 2:**
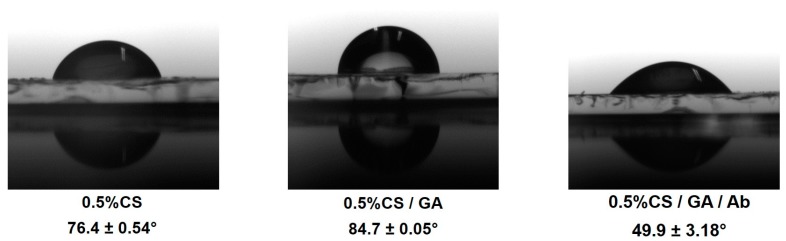
Measurements of contact angles of a water droplet on a 0.5% chitosan (CS) layer, crossed linked with glutaraldehyde (GA), and after immobilization of anti-OV6 antibodies.

Chitosan is a hydrophilic substance and its wettability can be affected by physical or chemical crosslinking. Glutaraldehyde (GA) is an organic crosslinking agent that normally increases the mechanical strength but reduces the hydrophilicity of the chitosan membrane [38]. Figure 2 shows that the contact angle of 0.5% un-crosslinked chitosan films was 76.4 ± 0.54° and increased to 84.7 ± 0.05° for crosslinked chitosan films. This result may be attributed to a reduction of hydrophilic groups (-OH or -NH_2_) in the membrane due to the reaction of the functional groups (-OH or -NH_2_) of chitosan and the aldehyde groups (-CHO) of GA, which makes chitosan film more hydrophobic and consists of long hydrophobic alkyl chains [39,40]. It has been reported that a hydrophobic surface yields higher affinity for functionalized adsorption of protein or antibody than a hydrophilic surface [41]. When the substrate was functionalized with the antibody, the contact angle was reduced to 49.9 ± 3.18° indicating hydrophilic surfaces, which might enhance cell attachments.

### 3.2. Detection of the OV6 Marker in Human Cancer Cell Lines via Flow Cytometry

**Figure 3:**
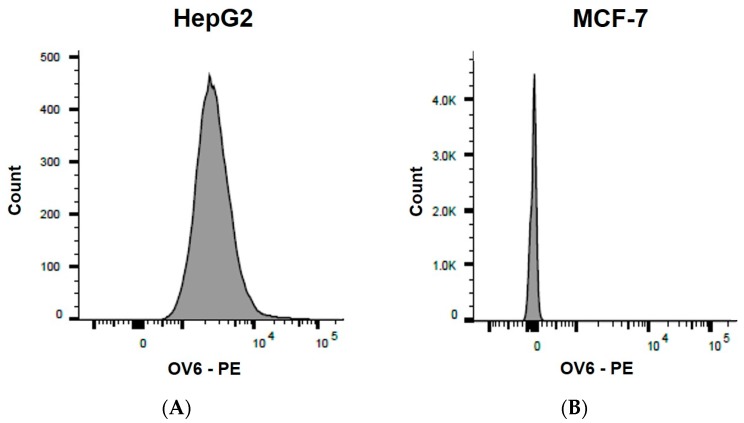
Flow cytometry detection of OV6 in HepG2 and MCF-7 cell lines. The figure shows OV6 + (positive) on HepG2 (**A**) and OV6- (negative) on MCF-7 (**B**).

The flow cytometry technique was conducted to confirm the presence of HOC in the HepG2 cell line that highly expresses the OV6 marker. Because breast cancer stem cells lack this surface marker, the MCF-7 cell line was used as a negative control (Figure 3).

Flow cytometry showed positive expression of the OV6 marker in hepatic cancer cells but not in breast cancer cells. The fluorescence intensity was higher in the hepatic cancer cell line (HepG2) than in the negative control breast cancer cell line (MCF-7). These results indicate that OV6 is a discriminating marker of hepatic cancer stem cells.

### 3.3. Electrochemical Behavior of the Fabricated Biosensor

**Figure 4:**
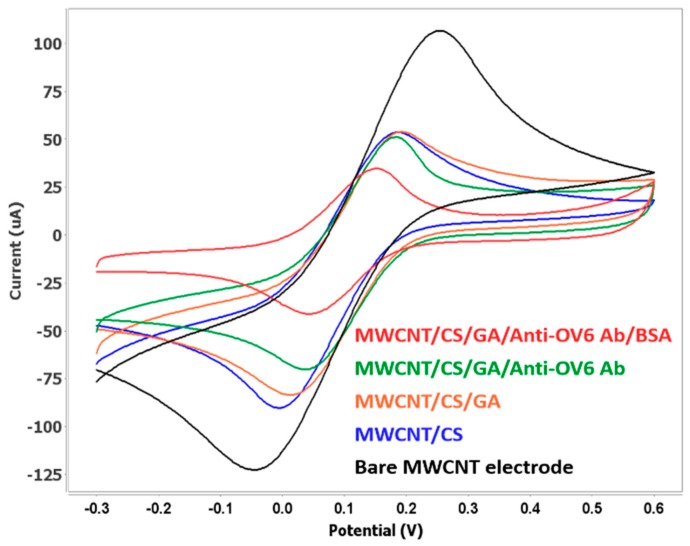
Cyclic voltammetry spectra of the functionalized MWCNT/CS/GA/anti-OV6 Ab/BSA electrode at a scan rate of 50 mV/s.

Electrochemical analysis has many applications for biomedical sensors and is a very effective tool for detecting changes in surface characteristics. Cyclic voltammetry is a convenient electrochemical method that is widely used to monitor the features of modified sensor surfaces [9,42]. In this work, to confirm the successful functionalization of the fabricated sensor on the MWCNT electrode, CV measurements were conducted after each electrode modification step, and the results are presented in Figure 4. The CV behavior was recorded in the potential range of−0.3 to 0.6 V in 10 mM Fe(CN_3_)^3−^ containing 100 mM KCl as a supporting electrolyte, and monitored for bare MWCNT, MWCNT/0.5%CS, MWCNT/CS/GA, MWCNT/CS/GA/anti-OV6 Ab, and MWCNT/CS/GA/anti-OV6 Ab/BSA electrodes. The current response decreased noticeably after each fabrication step of the biosensor.

The significant decrease in the peak current for MWCNT/CS compared to that of the bare MWCNT electrode indicates the formation of an insulation layer by chitosan that decreases the electron transport during oxidation and reduction processes. The further reduction in the CV current peak for MWCNT/CS/GA/anti-OV6 Ab confirms the immobilization of anti-OV6 antibodies. Subsequently, a 1% BSA solution was used to block the residual active carboxyl groups on the surface, which resulted in a decrease in the peak current due to the steric hindrance of BSA molecules in electron transfer. These changes in the electron transfer rate at each step indicate the successful fabrication of the electrochemical immunosensor.

### 3.4. Detection of Hepatic Oval Cells on the Fabricated Sensor

**Figure 5:**
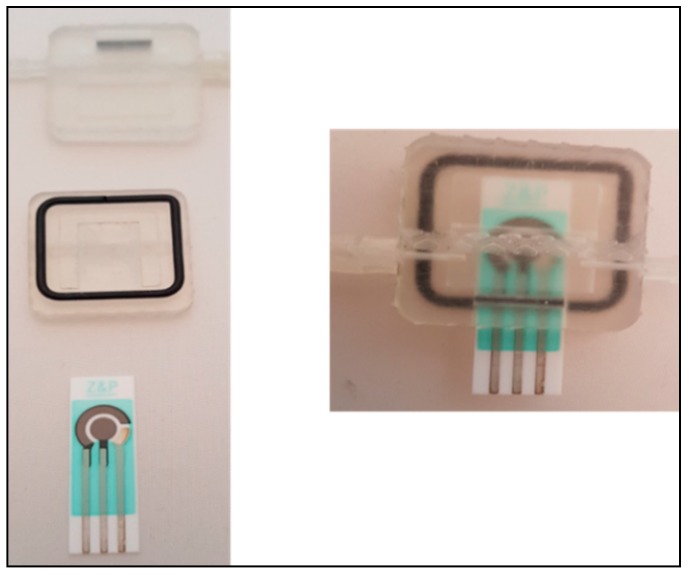
3D-printed flow cell parts before and after hand-assembly with the functionalized electrochemical sensor.

The HepG2 human liver cancer cell line was chosen in this study because it most resembles HCC and has the highest correlation of gene expression between HCC tumors and HCC cell lines [43]. However, it has been demonstrated that the HepG2 cancer cell line contains CSCs that can be identified by several cell surface markers, and OV6 is considered the best available marker of hepatic stem cells [44,45]. Due to the small number of CSCs and the difficulty of isolating these cells in a sample, a continuous flow system may enhance the capturing efficiency by allowing for continuous exposure of the sample to the functionalized sensor. Therefore, in this work, a disposable functionalized screen-printed electrode was embedded into a 3D-printed flow cell (Figure 5) and connected to a flow control system that allows continuous injection of cancer cell line as shown in Figure 1.

**Figure 6:**
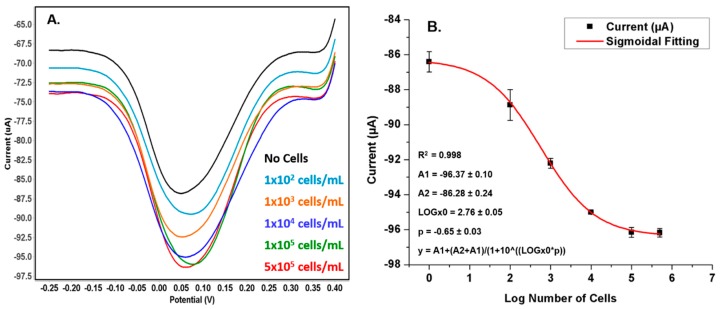
(**A**) Square wave voltammograms of the functionalized MWCNT/CS/GA/anti-OV6 Ab/BSA electrode with different number of HepG2 cancer cells contain hepatic oval cells (HOCs). (**B**) Plot of peak current response versus log number of hepatic cells and sigmoidal fitting. Potential step: 5 mV; amplitude: 25 mV; frequency: 15 Hz.

The specificity of the developed MWCNT/CS/GA/anti-OV6 Ab electrode to HOC was proven by the SWV results (Figure 6). The oval cells in the HepG2 cell line were captured by the anti-OV6 antibodies immobilized on the chitosan film. Treatment of the developed sensor using the HepG2 cell line dramatically changed the interfacial electron transfer resistance because captured cells blocked direct access of the ions to the electrode surface. The experimental results reveal well-defined voltammetric peaks that can be observed even at a low cells number (100 cells/mL). The SWV spectra show that the peak current increases negatively with increasing cell numbers. The changes in peak values indicate more coverage of the electrode surface by specifically captured cells. The SWV measured for the hepatic cells compared a blank test (no cells) to increased cell numbers, namely, 1 × 10^2^, 1 × 10^3^, 1 × 10^4^, 1 × 10^5^, and 5 × 10^5^ cells/mL, which took into consideration the limited number of stem cancer cells in each sample. The current response changed with the increase in the number of cells captured on the sensor. The dependency of current signal on the cell intensities may attributed to a electrochemical catalytic (EC’) mechanism. Figure 6B shows that the proposed sensor can detect HOCs in HepG2 cell line from 1 × 10^2^ to 5 × 10^5^ cells/mL range following sigmoidal fit. The reproducibility is shown as error bars, and electrodes exhibit a correlation coefficient of 0.998. It is well known that the MWCNT-modified electrode surface enhances electro-catalytic activity and molecule immobilization on functionalized surfaces. Indeed, modifying the MWCNT electrode with a chitosan film improves sensor stability and peak shape [31,32,33,34]. All these advantages with the high surface area possibly enhanced the sensitivity of the developed sensor to detect HOCs, which were present in a small number in the sample.

In previous work [9], an acoustic sensor was successfully developed to detect the CD133 marker on liver cancer stem cells. A quartz crystal microbalance with a dissipation monitoring (QCM-D) technique allows for a continuous flow of sample or buffer through the chamber and for control of the rate. However, the efficiency of the fabricated biosensor to detect the target cells was limited. By contrast, electrochemical sensors provide an alternative solution that has shown high sensitivity. Hence, in this work, the modified electrode was embedded with a 3D-printed flow cell to mimic the continuous flow chamber, which provides a promising strategy to enhance the capture efficiency of rare cells.

**Figure 7:**
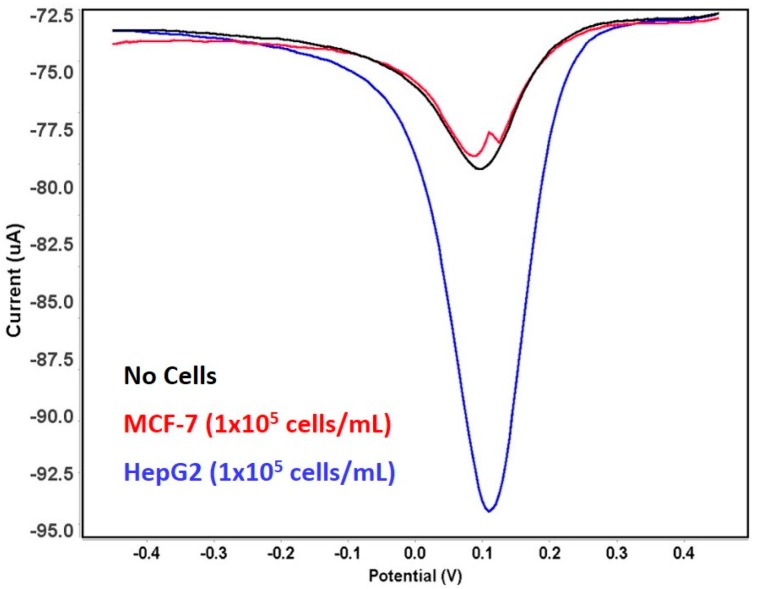
Square wave voltammograms of the developed sensor as a function of buffer (co cells) (black line), liver (HepG2) contains HOCs (blue line), and breast (MCF-7) (red line) cancer cell lines. Potential step: 5 mV; amplitude: 25 mV; frequency: 15 Hz.

To examine the specificity of the proposed sensor for OV6 detection, the functionalized electrode was incubated with a breast cancer cell line (MCF-7) that lacks the OV6 marker on the cell surface as confirmed by the flow cytometry analysis (Figure 7). No remarkable change of electrochemical current was observed when breast cancer cells (1 × 10^5^ cells/mL) were incubated with the proposed sensor due to the lack of OV6, which prevents the cells from being captured on the modified electrode. The clear difference in the peaks for hepatic and breast cancer cells indicates that the designed sensor can efficiently distinguish the cells that express OV6, although MCF-7 cells exhibited a small change of electrochemical signal that can be attributed to non-specific adsorption.

## 4. Conclusions

In summary, this work provides a promising strategy for diagnostic purposes that allows the use of disposable electrodes and exploits a 3D-printed flow cell for continuous exposure of the sample to the functionalized sensor. The developed electrochemical sensor is efficient in detecting hepatic oval cells via recognition of the OV6 marker and in discriminating between liver and breast cancer cells that lack this surface marker. The chitosan-modified MWCNT sensor is an effective, non-toxic, selective and sensitive sensing platform with a simple preparation process. The developed sensor can detect highly expressed markers on the cancer cell surface with good sensitivity due to the synergistic effects of MWCNT and chitosan that enhance electron transfer. This electrochemical method could be further exploited with other biomarkers to detect more tumor types and may be helpful for point-of-care diagnostics, monitoring the spread of tumors and assessing the response to therapy.
